# Antenatal care and mothers’ education improved iron-folic acid adherence at Denbiya district health centers, Northwest Ethiopia: using pills count method

**DOI:** 10.1186/s13690-019-0356-y

**Published:** 2019-06-25

**Authors:** Missa Tarekegn, Mamo Wubshet, Azeb Atenafu, Terefe Derso, Abere Woretaw

**Affiliations:** 1 Kolladiba Health center, Maternal and child health department, Gondar, Ethiopia; 2 Paulos Medical College Addis Ababa University, Addis Ababa, Ethiopia; 3 Department of Human Nutrition, Institute of Public Health, College of Medicine and Health Sciences, University of Gondar, Gondar, Ethiopia; 4 Department of Medical Nursing, School of Nursing, College of Medicine and Health Sciences, University of Gondar, Gondar, Ethiopia

**Keywords:** Iron, Folic acid, Adherence, Compliance, Pregnant women, Antenatal care

## Abstract

**Background:**

Anemia is the leading public health problem among pregnant women worldwide. Iron-Folic Acid (IFA) supplementation is the strategy to control pregnancy induced anemia, but its adherence status was not well studied.

**Objective:**

The aim of this study was to assess the prevalence of IFA adherence and associated factors among pregnant women attending antenatal care at Denbiya district health centers.

**Methods:**

Cross -sectional study design was conducted in Denbiya district health centers from April 2 to May 27, 2016. A total of 395 study participants were enrolled in the study. Systematic random sampling was used to select study participants. Data were collected using the interviewer-administered technique. Adherence to IFA supplementation was assessed by the pills count method. A logistic regression model was used.

**Results:**

The study revealed that the prevalence of good adherence towards IFA supplementation among Antenatal care (ANC) service users’ at Denbiya district health centers were found to be 28.01% [95% CI, 24.01, 35.9]. Attending secondary school and above [Adjusted Odds Ratio (AOR) = 3.44, 95% CI: 1.09, 10.92], having two ANC visits [AOR = 2.53, 95% CI: 1.34, 4.76] and three and above ANC visits [AOR = 4.14, 95% CI: 2.14, 8.01] were significantly associated with good adherence of IFA supplementation. To the contrary, husband education status; secondary school and above reduced the odds of good adherence by 77% compared to illiterates to IFA supplementation [AOR = 0.23, 95% CI: 0.07, 0.72].

**Conclusion:**

The prevalence of good adherence among pregnant women towards IFA supplementation was low. Mothers’ education and having two or more ANC visits were positively associated with good adherence towards IFA supplementation.

## Background

Anemia is the leading public health problem worldwide. Globally, the estimated prevalence of anemia was 24.8% in the general population; 47.4% in preschool-aged children, 41.8% in pregnant women, and 30.2% in non-pregnant women [1].

Pregnant women are at high risk of iron deficiency anemia due to increased nutrient requirements. As a result, 38.2% of pregnant women suffered from anemia worldwide [2]. Inadequate dietary intake, previous pregnancy, normal recurrent loss of iron from menstrual blood, morning sickness, and previous anemia history were the risk factors of anemia for pregnant women [3]. Prophylaxis IFA supplementation is an important option to prevent iron deficiency anemia in pregnant women [4]. IFA supplementation is part of Antenatal Care (ANC) to reduce the risk of low birth weight, maternal anemia, and iron deficiency [5]. In South India, the IFA adherence rate was 64.7%. The effectiveness of IFA supplementation depends on the adherence or compliance of pregnant women. In Muntinlupa, Philippine, the compliance rate of pregnant mothers to IFA is 54% [6]. In Brazil, the IFA adherence rate of pregnant women is 82%, but its adherence rate decreased as the frequency of doses increases [7]. Forget fullness, vomiting, nausea, heartburn, and diarrheas were the main reasons of drug interruption [8–10]. In Mozambique, the iron sulfate adherence was 79% for having two or more visits, 53% for those having adequate prenatal care, and 67% for those who complete their intake of IFA tablets [11]. In Tigray, Ethiopia the rate of adherence to IFA supplementation was 37.2% [12] and Addis Ababa 60% [13]. The presence of iron sulfate side effects such as constipation, darkened or green stools, diarrhea, loss of appetite, nausea, stomach cramp, and vomiting decrease the IFA adherence rate in pregnant women [7, 14]. Conducting research on IFA adherence for pregnant women provides a double benefit for the mother and the fetus [15]**.** Iron-folic acid supplementation is one of a strategy made to prevent anemia for pregnant women [4, 5], but there is no evidence on the adherence of IFA supplementation among pregnant women in the study area. Therefore, the present study determines the adherence to IFA supplementation among pregnant women.

## Methods

### Study setting and period

Cross-sectional study was conducted at Dembiya district health centers (Guramba, Kolladiba, Robit, and Chuahit) among pregnant women from April 2 to May 27, 2016. These health centers are found in the North Gondar zone of Amhara regional state of Ethiopia. Dembia district is situated around 772 Km away from Addis Ababa (the capital city of Ethiopia). These health centers serve for more than half a million of people in the town and rural areas. At the moment there were around 789 pregnant women in all of the health centers.

### Sample size determination and sampling procedures

All pregnant women who had ANC follow up in the Denbiya district health centers were a source of population. Pregnant women who received IFA supplementation for at least one month prior to data collection were included in the study. The sample size was determined using a single population proportion formula through the EPi Info Stat Calc Program with the assumption of 95% level of confidence, 5% marginal error, and taking the prevalence of IFA adherence in Tigray, Ethiopia (37.2%) [12]. After computing the calculation, the sample size was 359. Finally, considering a10% non-response rates, the final sample size was 395. The sampling frame was developed according to the order of the pregnant women attending the ANC clinic. A systematic random sampling technique was used to select study participants by calculating a sampling interval as the total population to sample size. Then each participant was selected every two-person pattern.

### Data collection tools and procedures

The structured interviewer-administered questionnaire which is composed of socio-demographic and obstetric history were used to collect the data [12, 13]. Besides, data on adherence of IFA was collected using pills count methods. Data were collected by three midwives (one supervisor and two data collectors). To maintain the quality of the data, the questionnaire was pretested and half day training was given to data collectors and supervisors. The questionnaire first developed in English and translated to Amharic local language to collect data then back to English for analysis to maintain its consistency.

### Operational definitions

#### Antenatal care visit

Antenatal care provided by skilled health personnel (doctor, nurse or midwife) during pregnancy.

#### Trimester

The number of weeks during pregnancy (1st, 1-12 weeks, 2nd, 13–26 weeks, and 3rd, 27-40 weeks).

#### Gravidity

The number of pregnancy whatever the outcome.

#### Parity

The number of live births among pregnancy.

#### IFA adherence

Good Adherence was considered as a pregnant woman who took ≥65% of the total prescribed IFA supplementation per month; whereas the opposite is true for non-adherence [12].

#### Data processing and analysis

Data were entered into the EPi Info Version seven and exported to SPSS Version twenty for analysis. Descriptive statistics, such as means, medians, frequencies, and percentages were calculated. All variables having *p*-value ≤0.2 in bivariate analysis entered into the multivariate logistic regression model to identify the effect of the independent variable on the outcome variables. *P*-value < 0.05 was considered statistically significant and AOR with a 95% CI was calculated to see the presence of associations. Model fitness was checked by using Hosmer and Lemeshow goodness of fit test.

## Result

### Socio-demographic characteristics of pregnant women

A total of 395 pregnant women were enrolled in the study with a response rate of 96.7%. More than one-third (43.2%) of the pregnant women were found to be in the age range of 25-31 years. Majorities (96.6%) were married, and 84% were housewife. Nearly two-thirds (59.2%) of pregnant women were illiterate (Table 1).

**Table 1:** Socio demographic characteristics of pregnant women attending ANC service at Denbiya district health centers, Northwest Ethiopia, 2016 (*n* = 382)

Variables	Frequency (*n*)	Percent (%)
Age in years		
18–24	79	20.7
25–31	165	43.2
32–42	138	36.1
Marital status		
Married	369	96.6
Unmarried	13	3.4
Religion		
Orthodox	357	93.5
Muslim	23	6
Protestant	2	0.5
Ethnicity		
Amhara	372	97.4
Tigray	10	2.6
Mother’s education status		
Illiterate	226	59.2
Primary school	83	21.7
Secondary school and above	73	19.1
Husband education status		
Illiterate	209	54.7
Primary school	97	25.4
Secondary school and above	76	19.9
Occupation		
Housewife	321	84
Government employee	23	6
Private employee	11	2.9
Merchant	27	7.1
Monthly household income in Ethiopia		
Birr (EB)		
< 1000	115	30.1
1000–2000	114	29.8
2001–3000	95	24.9
> 3000	58	15.2

### Obstetric and health-related characteristics of pregnant women

One hundred fifty-four (40.3%) of pregnant mothers had two ANC visits. Nearly two-thirds (62%) were multigravida, and (57.3%) were ≤ two trimesters (Table 2).

**Table 2:** Obstetric characteristics of pregnant women attending ANC service at Denbiya district health centers, Northwest Ethiopia, 2016 (n = 382)

Variables	Frequency (*n*)	Percent (%)	
Number of ANC visit			
One times	119	31.2	
two times	154	40.3	
Three and above	109	28.5	
Parity			
Nulliparous	58	15.2	
Primiparous	84	22	
Multiparous	240	62	
Gravidity			
Primigravida	53	13.9	
Multigravida	329	86.1	
Trimester			
≤ two	219	57.3	
three	163	42.7	
Acute illness			
Yes	66	17.3	
No	316	82.7	

### Prevalence of IFA adherence among pregnant women

The prevalence of good adherence among pregnant women towards IFA supplementation was found to be 28.01% [95% CI, 24.01, 35.9]. The mean (SD) IFA consumption among pregnant women was 1.08 ± 0.24 pills.

**Fig. 1:**
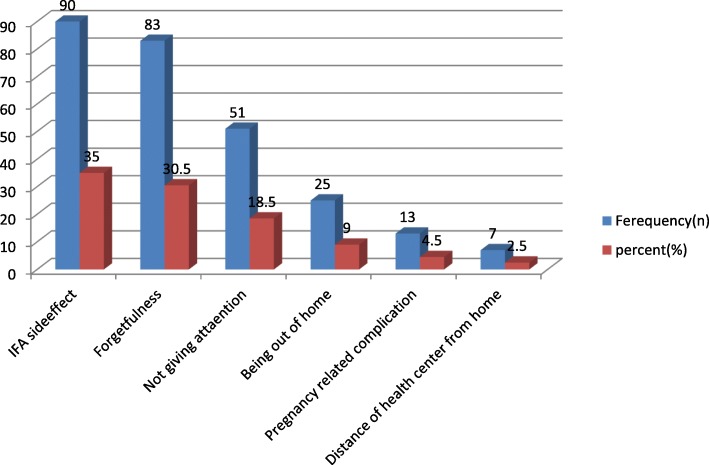
Reasons for IFA non-adherence among pregnant women attending ANC service at Denbiya district health centers, Northwest Ethiopia, 2016

Gastrointestinal discomfort due to side effect 35%, forgetting 30.5%, and not giving attention 18.5% are the reasons of non-adherence to IFA supplementation (Fig. 1).

### Factors associated with IFA adherence among pregnant women

In both the bivariate and multivariable analysis; maternal education and numbers of ANC visits were significantly associated with good adherence to IFA supplementation. Education status; secondary school and above [AOR = 3.44, 95% CI: 1.09, 10.92], and antenatal care visits; two ANC visit [AOR = 2.53, 95% CI: 1.34, 4.76], and three and above ANC visit [AOR = 4.14, 95% CI: 2.14, 8.01] were significantly associated with good adherence to IFA supplementation. Whereas husband education status; secondary school and above reduced the odds of good adherence by 77% compared to illiterates to IFA supplementation [AOR = 0.23, 95% CI: 0.07, 0.72] (Table 3).

**Table 3:** Factors associated with IFA adherence among pregnant women attending ANC service at Denbiya district health centers, Northwest Ethiopia, 2016 (*n* = 382)

Variables			Crude OR	Adjusted OR
	Adherence status		95% CI	95% CI
	Good	Poor		
Mother ‘s education status				
Illiterate	63	163	1	1
Primary school	24	59	1.05 (0.603,1.836)	1.76 (0.88,3.50)
Secondary school and	20	53	0.98 (0.54,1.76)	3.44 (1.09,10.92)*
above				
Husband education status				
Illiterate	63	146	1	1
Primary school	26	71	0.85 (0.50,1.45)	0.60 (0.31,1.16)
Secondary school and	18	58	0.72 (0.39,1.32)	0.23 (0.07,0.72)*
above				
Number of ANC visit				
One times	19	100	1	1
two times	45	109	2.17 (1.19,3.96)	2.527 (1.341,4.761)*
Three and above	43	66	3.43 (1.84,6.39)	4.14 (2.14,8.01)*
Trimester				
≤ two	50	169	1	1
three	57	106	1.82 (1.16,2.85)	1.16 (0.62,2.17)

*p-value < 0.05

## Discussion

The prevalence of good adherence among pregnant women towards IFA supplementation was found to be 28.01% [95% CI, 24.01, 35.9]. The finding of the current study is higher than studies conducted in Afar, Ethiopia (22.9%) [12] and Mecha, Ethiopia (20.4%) [16]. To the contrary, the finding of this study is much lower than studies conducted from Addis Ababa, Ethiopia (60%) [13], Mizan Tepi, Ethiopia (70.6%) [17], Eritrea (64.7%) [18], Enugu, Southeastern Nigeria (64.5%) [19], Senegal (69%) [20], Kathmandu, Nepal (73.2%) [21], and Iraq (51.47%) [22]. The possible discrepancy might be due to variations in socio-demographic characteristics, data collection tools and the cut point of adherence status, and period of data collection.

The current study revealed that maternal education is an independent factor of good adherence to IFA supplementation. Pregnant mothers who had secondary education and above were 3.44 times more likely [AOR = 3.44, 95% CI; 1.09, 10.92] to have good adherence towards IFA supplementation compared to those had no secondary education and above. This finding is supported by studies from Addis Ababa, Ethiopia [13], Asela, Ethiopia [10], West Iran [23], and West Bengal, India [24]. This might be due to education is more likely to enhance pregnant women awareness on the outcome of IFD deficiency and ways to overcome these deficiencies from different sources including advice from health workers. This implies that educated women have a greater ability to stick to health care inputs such as IFA which offer better for fetal growth and development; and care for both the infant and the mother [25, 26].

Numbers of ANC visits are found to be the determinant factors of good adherence towards IFA supplementation. Pregnant mothers who had two ANC visits are 2.53 times more likely [AOR = 2.527, 95% CI; 1.341, 4.761] to have good adherence towards IFA supplementation compared to those who had one ANC visit. Similarly, Pregnant mothers who had three and above ANC visits are 4.14 times more likely [AOR = 4.14, 95% CI; 2.14, 8.01] to have good adherence towards IFA supplementation compared to those who had one ANC visit. This finding is supported by studies Eritrea [18], Kiambu, Kenya [27], and Northwest Ethiopia [28]. This is the fact that ANC is the vital route for the delivery of iron supplementation and reinforcement of adherence. This suggests increase ANC visits are a good opportunity to increase contact between pregnant women and health professionals. Thus, health professionals can disseminate key information/messages, especially the benefits of IFA supplementation.

Husband education status; secondary school and above reduced the odds of good adherence by 77% compared to illiterates [AOR = 0.23, 95% CI: 0.07, 0.72]. This might be due to the misunderstandings/misperception of educated husbands on side effects and may think IFA has a negative adverse effect on the fetus like the other unsafe drugs. This suggests that educated husbands may affect the autonomy of the mothers to take iron-folic acid supplementation. The relationship between IFA adherence and husbands’ education needs further investigation, triangulated with a quantitative and qualitative study.

### Limitations

The study did not assess the level of adherence and some other factors like knowledge of anemia. Further studies are recommended with all the necessary variables with strong study designs triangulated with qualitative methods.

## Conclusions

The prevalence of good adherence among pregnant women towards IFA supplementation is low. Mothers’ education and two or more ANC visits are positively associated with IFA adherence. The IFA side effect was the main reason for pregnant women not taking regularly. Hence, promoting the benefits of frequent ANC, provide information about the benefits of IFA, side effect, and the effect of micro-nutrient deficiency should be intensified. Health professionals at the health facility should sensitize pregnant women on the need to continuously take the supplements throughout pregnancy. Local health extension workers, husbands and generally community should also be strongly involved in the promotion of prenatal iron supplementation.

## Data Availability

The datasets supporting the conclusion of this article are included in this article.

## Abbreviations

ANC: Antenatal care
AOR: Adjusted odds ratio
CI: Confidence Interval
COR: Crude Odds Ratio
IFA: Iron-folic acid
